# Effect of Surface Modification of a Dental Composite on the Adhesion of *Streptococcus mitis*, *Streptococcus mutans*, and *Candida albicans*: An In Vitro Study

**DOI:** 10.3390/pathogens14090909

**Published:** 2025-09-10

**Authors:** Santiago Arango-Santander, Claudia María Bedoya-Correa, Camila Soto, Santiago Bustamante, John Franco

**Affiliations:** GIOM Group, Faculty of Dentistry, Universidad Cooperativa de Colombia, Medellin 055421, Colombia; claudia.bedoyac@campusucc.edu.co (C.M.B.-C.); camila.soto@campusucc.edu.co (C.S.); santiago.bustamante@campusucc.edu.co (S.B.); john.francoa@campusucc.edu.co (J.F.)

**Keywords:** surface modification, bacterial adhesion, fungal adhesion, biomimetics, soft lithography, *Streptococcus mutans*, *Streptococcus mitis*, *Candida albicans*

## Abstract

Adhesion of different microorganisms to the surface of dental materials has generated significant interest since one of the most important requirements of biomaterials to be considered successful is their ability to withstand the damage caused by microorganisms that may lead to failure and the onset of different pathologies, such as caries. In vitro testing has demonstrated that surface modification is an alternative approach to reduce the adhesion of microorganisms to surfaces. The objective of this work was to assess the adhesion of *Streptococcus mitis*, *Streptococcus mutans*, and *Candida albicans* to a dental composite surface modified following a biomimetic approach and coated with salivary proteins. Soft lithography was used to copy the topography from the *Crocosmia aurea* leaf and then transfer it to the surface of dental composite discs that were coated with saliva proteins. Surfaces were characterized by contact angle and atomic force microscopy. *S. mitis*, *S. mutans*, and *C. albicans* were used to assess bacterial and fungal adhesion in monoculture and co-culture. The topographic modification of the surface of a dental composite reduced the adhesion of assessed microorganisms and the adhesion of these species in monoculture and co-culture on saliva-coated surfaces was higher than on topographically modified surfaces.

## 1. Introduction

Adhesion of microorganisms to the surface of dental materials has gained significant interest in recent decades due to the fact that these materials must perform successfully in the oral cavity while subjected to a wide variety of bacteria, yeasts, and viruses that may adhere to their surfaces and cause deterioration and failure, leading to the occurrence of different conditions, including dental caries or periodontal disease [1,2]. In addition, dental materials inside the oral cavity are coated with a thin layer of saliva that contains proteins, which are adsorbed onto their surfaces and facilitate the adhesion process [3]. The formation of this layer, known as the acquired pellicle (AP), is the first stage that occurs on a clean dental surface. As mentioned, proteins forming the AP adsorb onto the surface of natural and artificial dental materials and create ideal conditions for microorganisms to adhere. One of the first microorganisms to arrive and initiate the process of adhesion and colonization is *Streptococcus mitis*, followed by other streptococcal species and Gram-positive bacilli [4,5,6,7]. *S. mitis*, a commensal, acid-sensitive species, adhere to the surface, assisted by salivary proteins, is considered as an accessory pathogen due to its ability to form multispecies biofilms increasing the virulence of a polymicrobial consortium [8,9]. As different micro-organisms arrive, including highly acidogenic species such as *Streptococcus mutans* and commensal *Candida albicans*, a more specific adhesion process begins, and a more mature biofilm will be formed in the following hours [7,8,10,11]. *S. mutans* overgrowth causes an imbalance in the biofilm due to its ability to produce high amounts of glucans, synthesize organic acids, and survive in harsh environments making it cariogenic [8,12], while *C. albicans* is able to change the morphology from yeast to a filament structure, which is essential for its pathogenicity, biofilm formation, and establishment of polymicrobial interactions [12,13], thus potentializing the formation of this cariogenic biofilm [14]. This oral biofilm will cause the onset of different conditions, dental caries being the most predominant, that will affect the health status of patients [4,15,16].

The adhesion process is also dependent on the surface characteristics of the materials, such as hydrophobicity, roughness, or chemical composition. The environment also provides some factors, including the temperature, availability of nutrients, the presence of other colonizers, the presence and concentration of harmful substances (antimicrobial compounds, metabolic subproducts, or ions, among others), and the presence of cells from the host’s immune system [16].

Many approaches have been investigated to reduce the adhesion of microorganisms to the surface of biomaterials. In dentistry, the most common approach is brushing and flossing followed using antimicrobial chemical compounds, such as chlorhexidine or fluoride [15]. However, these methods are not sufficient, and other approaches have been explored. Surface modification is an alternative strategy that has been investigated during the last two decades and has shown remarkable results at reducing the adhesion of different microorganisms to the surface of medical and dental biomaterials in vitro [17,18]. Surface modification may be broadly classified as chemical or physical (topographical). The latter intends to modify the surface topography of a material by additive or sub-tractive methods [16,17,18,19,20]. One of such methods, known as soft lithography, relies on using a master model that is copied using an elastomer. This copied topography is then transferred to another surface using a variety of compounds. The master model may be fabricated using techniques such as photolithography or may be copied from natural (animal or vegetal) surfaces, an approach known as biomimetics [18,19]. Surfaces modified using this approach have shown promising in vitro results [17,18,19]. However, most investigations have focused on analyzing the behavior in models of a single bacterial species when in contact with modified surfaces. Moreover, studies on dental materials have not addressed the use of salivary proteins attached to the surface of these materials before testing the antiadhesion capabilities. Therefore, the objective of this work was to assess the effect of topographic surface modification of a dental composite coated with salivary proteins on the adhesion of oral microorganisms following a biomimetic approach. The hypothesis of the study was that topographic modification of the surface characteristics of the composites reduces microbial adhesion regardless of the analyzed microorganisms.

## 2. Materials and Methods

### 2.1. Sample Preparation

This quantitative, experimental, in vitro study was approved by the Ethics Committee from Universidad Cooperativa de Colombia (Act 003, 2022). Dental composite discs (10 mm diameter and 2 mm thickness, Spectra Basic, Dentsply Sirona, Charlotte, NC, USA) were fabricated using an acrylic template to ensure homogeneity. The template was placed on a glass slab, the composite was added to each hole in the template, a Mylar strip (New Stetic, Guarne, Colombia) was placed on top of the composite and another glass slab was placed to photopolymerize the composite for 40 s (20 s per side) using a calibrated LED dental photopolymerization unit (Woodpecker, Guilin, China). Each disc was finished with Sof-Lex discs (3M ESPE Dental products, Saint Paul, MN, USA) by a single calibrated operator following a unidirectional strategy. Four polishing discs (from 100 μm to 8 μm) were used with a dental handpiece (NSK, Nakanishi Inc., Tochigi, Japan) at 15,000 rpm, followed by final polishing with 1.0 μm diamond paste (Leco Corporation, St. Joseph, MI, USA) and 0.5 μm silica (Leco Corporation, St. Joseph, MI, USA). Composite discs were then sequentially cleaned in an ultrasonic bath using 99.8% acetone (Merck Millipore, Burlington, MA, USA), distilled water (Protokimica, Medellín, Colombia) and 99% ethanol (Merck Millipore, Burlington, MA, USA). 192 composite discs were obtained and allowed to dry in the air and were divided into four groups described in Section 2.2. Sample calculation was performed by convenience sampling to 48 discs per group for the adhesion tests.

### 2.2. Master Model and Soft Litography

**Figure 1:**
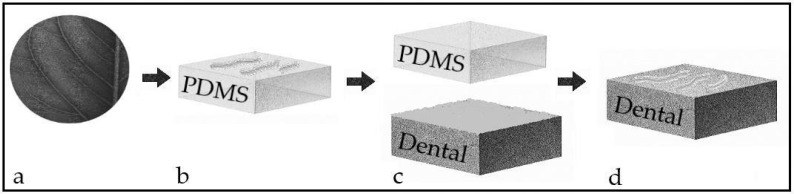
Schematics of the soft lithography process. The topography from the *C. aurea* leaf (**a**) is copied using PDMS (**b**). A dental composite adhesive is applied on a dental composite surface and the PDMS stamp is brought into contact with the dental composite surface (**c**). After polymerization of the dental adhesive, the topography from the *C. aurea* leaf is transferred to the surface of the dental composite (**d**).

The lamina of the *Crocosmia aurea* leaf was used to fabricate the master model. Such leaf was selected due to its high hydrophobicity and self-cleaning properties at simple observation. The leaf was cut into 5.0 cm-diameter segments. Each segment was bonded to the bottom of a silicone container with the lamina facing upward. Polydimethylsiloxane (PDMS, Silastic T-2, Dow Corning Corporation, Midland, MI, USA) was prepared following the manufacturer instructions and poured on each container until covering the leaf segment. A 24-h polymerization time, followed by a 3-h heat polymerization time at 80 °C, was allowed. After PDMS polymerization, a stamp containing the embedded topography from the leaf was obtained. To transfer the embedded topography onto the sur-face of the composite discs, a 50 μL drop of universal bond (3M ESPE Dental products, Saint Paul, MN, USA) was added to each disc. Then, the PDMS stamp was placed on the drop and gentle pressure was applied to ensure that the liquid bond covered the entire disc surface. Photopolymerization of the bond for 3 s with a LED lamp was performed. This process is depicted in Figure 1. The discs were then allocated to four groups: L-Sal (polished, coated with salivary proteins), L-SSal (polished, uncoated), P-Sal (modified, coated with salivary proteins), and P-SSal (modified, uncoated).

### 2.3. Saliva Collection and Treatment

15 mL of saliva stimulated by mastication of 1 g of sterile paraffin were collected. Saliva was acquired from a healthy individual who met the following requirements: no caries or periodontal disease, no systemic conditions, no smoking, and no pharmacological treatment. Mouthwash use was suspended 7 days before taking the sample, the subject performed a thorough brushing and flossing procedure after breakfast and no food was consumed one hour before collecting the saliva. According to Resolution 8430 (1993) of the Colombian Ministry of Health, this study was classified as “no risk” (Chapter 11) as it involves no intervention or modification of physiological variables. The study utilized a saliva sample provided by one of the authors to obtain proteins for coating composite discs. No saliva samples were collected from patients or other research participants. Per Chapter 16, Paragraph 1 of the Resolution, signed informed consent is not required for no-risk research.

In order to eliminate cell remnants or insoluble materials, the collected saliva was transferred to Eppendorf tubes and centrifuged at 12,000 rpm for 10 min at 4 °C (ThermoFisher Scientific, Waltham, MA, USA). Supernatants were transferred to fresh Eppendorf tubes and centrifuged following the same conditions. Then, the soluble fraction of the saliva was passed through a sterile 0.22 μm membrane syringe filter (New LBSSP E022, Jinan, China). This procedure was performed inside a vertical laminar flow chamber (BioBase, Jinan, China) to avoid cross-contamination. A sterility test was performed by inoculation of 100 μL of the previously filtrated saliva in brain-heart infusion (BHI) agar (Scharlab S.L., Barcelona, Spain) and incubated in microaerophilic conditions for 48 h. Sterile saliva was frozen at −20 °C.

The Bradford method with the Bio-Rad Protein Assay kit (Sigma Aldrich, Missouri, MO, USA) was used for total protein quantification. Before determining the total protein concentration in the sterile saliva, a calibration curve with standard bovine serum albumin (BSA) was performed. Then, 10 μL of sterile saliva was mixed with 200 μL of Coomassie G-250 bright blue stain (ThermoFisher Scientific, Waltham, MA, USA). Samples were incubated in the dark at RT for 5 min and absorbance at 595 nm optical density (OD) was measured.

Composite discs from L-Sal and P-Sal groups were placed at the bottom of a 96-well, flat-bottomed polystyrene plate (Costar, Corning Inc., New York, NY, USA). A 50 μL aliquot of sterile saliva was added to each disc. Incubation at 37 °C for 2 h, to allow salivary proteins to adsorb onto the disc surface, was performed. Then, the saliva was gently removed by pipetting without touching the disc surface and drying in air inside the flow chamber was allowed.

### 2.4. Surface Hydrophobicity and Roughness

Surface hydrophobicity of the experimental groups was assessed by the sessile drop method. A 4 μL drop of 0.9% saline (Corpaul, Medellín, Colombia) was placed on top of a disc from every group. 3 discs per group were evaluated. For image obtention, a camera with a 25 X macro lens (CoPedvic, Shenzhen, China) was used and the contact angle was determined using the AxioVision software (v4.9.1.0).

Surface roughness was assessed by atomic force microscopy using 10 µm × 10 µm images obtained with an atomic force microscope in tapping mode using a TAP300 tip (AFM, XE7, Park Systems, Suwon, Republic of Korea). The arithmetic average of the roughness profile (Ra) was calculated using the XEI V.4.1.0 software version (XE7, Park Systems).

### 2.5. Biological Evaluation

*Streptococcus mutans* (ATCC 25175, Microbiologics, St. Cloud, MN, USA), *Streptococcus mitis* (NCIMB 13770, Microbiologics, St. Cloud, MN, USA), and *Candida albicans* (ATCC 10231, Microbiologics, St. Cloud, MN, USA) were used. The strains were kept frozen at −20 °C in 20% glycerol (IBI Scientific, Dubuque, IA, USA). Before microbiological testing, *S. mutans* and *S. mitis* were reactivated in BHI agar (Scharlab S.L., Barcelona, Spain) and *C. albicans* was reactivated in Sabouraud Chloramphenicol agar (Scharlab S.L., Barcelona, Spain). The strains were then incubated at 37 °C in microaerophilic conditions (5% CO_2_) for 18 h. After reactivation of the reference strains, cell suspensions with the 18-h cultures were prepared by adding colonies to 10 mL of BHI broth (Merck KGaA, Darmstadt, Germany) supplemented with 5% saccharose (Fisher Chemical, Waltham, MA, USA). Continuous measurements with a turbidimeter (Velp Scientifica, Usmate, Italy) were performed until a 90 ± 5 NTU (Nephelometric Turbidity Unit), equivalent to 0.5 in McFarland standard was obtained, which corresponded to a cell concentration of 1.5–2.0 × 10^8^ CFU/mL (Colony Forming Units per milliliter). For co-culture adhesion tests, a 1.5–2.0 × 10^6^ CFU/mL cell concentration per strain was used.

### 2.6. Monospecies Adhesion Culture Test

Composite discs from the experimental groups (L-Sal, L-SSal, P-Sal, and P-SSal) were placed at the bottom of 24-well flat-bottomed polystyrene plates (Costar, Corning Inc., New York, NY, USA). 50 µL of each strain suspension (1.5–2.0 × 10^8^ CFU/mL) was independently placed on the surface of a composite disc. Plates were incubated at 37 °C in microaerophilic conditions (5% CO_2_) for 6 h to allow microbial adhesion. Then, the composite discs were removed from the plate and washed twice with 100 µL of 0.9% saline (Corpaul, Medellín, Colombia) to detach cells that did not adhere to the surface or that adhered poorly. Composite discs were then sonicated in 2 mL of 0.9% saline (Corpaul, Medellín, Colombia) using an ultrasonic sonicator (QSonica Q500, Newtown, CT, USA) with a 50% power and a 30-sec pulse to detach adhered cells. Microdilutions (10^−1^–10^−3^) from the sonicated products were performed and 10 µL of each dilution was inoculated onto BHI agar (Scharlab S.L., Barcelona, Spain) using the drop method. Cultures were incubated at 37 °C in microaerophilic conditions (5% CO_2_) for 48 h and counting of the viable cells was performed. For quantification of the number of microbial cells adhered to the experimental surfaces, results were expressed as Colony Forming Units per disc (CFU/disc) by the following equation:

(1) $$CFU/disc\;=\;number\;of\;colonies\;\times \;10^{n}\;\times \;100$$

where *n* corresponds to the absolute value of the dilution at which it is possible to observe between 30 and 300 colonies and 100 is the correction factor of the seeded volume.

### 2.7. Co-Culture Adhesion Test

This test was used to assess the adhesion ability of *S. mutans*, *S. mitis*, and *C. albicans* to modified and unmodified composite surfaces. A 1.5–2.0 × 10^6^ CFU/mL cell concentration per strain was obtained and a mixture of the strains in equal proportion (1:1:1) was prepared. Composite discs were placed at the bottom of 24-well flat-bottomed polystyrene plates (Costar, Corning Inc., New York, NY, USA). 50 µL of the microbial mixture was added to the surface of the composite discs and incubation at 37 °C in microaerophilic conditions (5%CO_2_) for 6 h was completed. After incubation, the discs were washed twice with 100 µL of 0.9% saline (Corpaul, Medellín, Colombia) and the same sonication, dilution, culture, and counting of viable cells processes reported in the monospecies test were performed. Two replicas were carried out and each replica was performed in quadruplicate for each experiment.

### 2.8. Statistical Analysis

A descriptive analysis of the microbial adhesion and contact angle variables according to the experimental treatments by estimating the summary measures (central tendency, dispersion, and position) was performed. The comparison between the results from microbial adhesion and contact angle was performed using the Kruskal–Wallis h test with multiple comparisons, the Mann–Whitney test with Bonferroni correction, and one-factor Anova with Tukey or Games-Howell HSD post hoc test. The comparison of the microbial adhesion in monoculture and co-culture was performed using the Mann–Whitney U test. For all analyses, a previous verification of normal distribution and variance homogeneity was performed using the Shapiro–Wilk and Levene tests, respectively. All analyzes were performed in IBM^®^ SPSS 29 software and a *p* value < 0.05 was considered as criterion for statistical significance.

## 3. Results

### 3.1. Surface Hydrophobicity and Roughness

**Figure 2:**
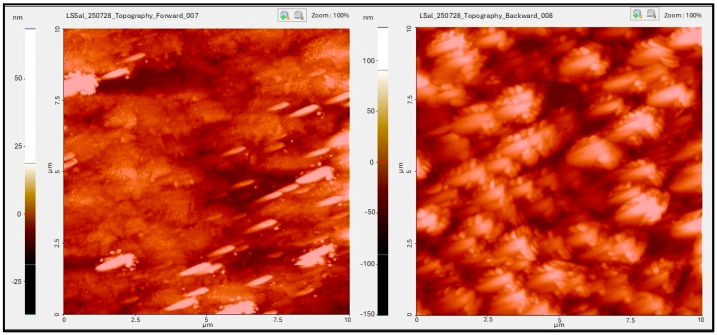
AFM images of uncoated (**left**) vs. salivary protein-coated (**right**) surfaces. Organized clusters in globular form are observed on the salivary protein-coated surface.

Deposition of the saliva proteins was successful, as shown in Figure 2.

**Figure 3:**
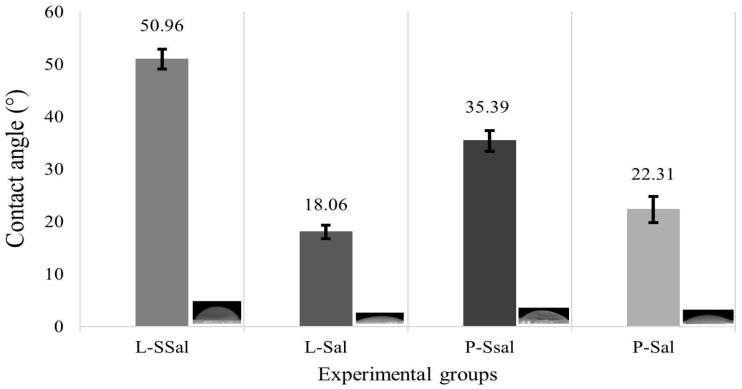
Contact angle values of the experimental groups: L-SSal (polished, uncoated), L-Sal (polished, coated with salivary proteins), P-SSal (modified, uncoated), P-Sal (modified, coated with salivary proteins). *p* value = 0.005.

The analysis of surface hydrophobicity showed the highest contact angle in the control group (L-SSal, 50.9 ± 1.9°) and the lowest in the L-Sal experimental group (18.06 ± 1.3°). The comparison in the contact angle averages among the experimental groups showed statistically significant differences in all categories (Anova *p* < 0.001, Figure 3). These results demonstrate that the topographic modification of the surfaces generates a significant reduction in the hydrophobicity and this effect is enhanced by the saliva coating, which suggests an increase in the affinity of these surfaces for aqueous media.

The smoothest surface was exhibited by the LSSal group (Ra: 0.066 µm) followed by the LSal (Ra: 0.378 µm) and the PSal (Ra: 0.474 µm) groups. The roughest surface was shown by the PSSal group (Ra: 1.975 µm).

### 3.2. Microbial Adhesion

Microbial adhesion tests were performed on the surface of 192 composite discs, 48 discs for each assessed category. All the discs from the L-Sal and P-Sal categories were coated with 0.139 mg/mL of total protein. The comparison of the adhesion to the different experimental groups showed that the adhesion of *S. mutans* and *S. mitis* in monoculture was significantly lower to the patterned discs (with and without saliva) when compared to the L-Sal group (Games-Howell *p* < 0.02). Similarly, the bacterial adhesion was significantly lower in the L-SSal group compared to the L-Sal group (Games-Howell *p* = 0.044). The adhesion of *C. albicans* in monoculture to the experimental discs did not show statistically significant differences (Anova *p* = 0.100, Table 1). However, the average number of CFU/disc was lower after comparison with *S. mutans* and *S. mitis*, strains that exhibited higher adhesion ability to surfaces.

Adhesion of *S. mutans* in co-culture showed significantly lower values on modified vs. polished discs (*p* < 0.02). When considering the polished discs, the adhesion to uncoated discs was lower than coated discs (*p* = 0.041). The adhesion of *S. mitis* in co-culture did not show statistically significant differences among the experimental discs (Kruskal–Wallis *p* = 0.661). The adhesion of *C. albicans* in co-culture showed significantly lower values to modified vs. polished discs coated with saliva proteins (*p* < 0.004). No statistically significant differences were observed in the modified discs (*p* = 1.000, Table 2).

**Figure 4:**
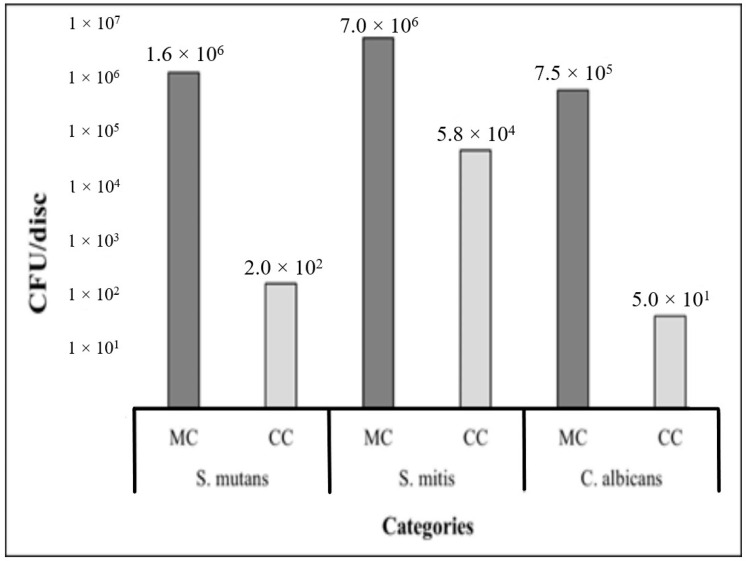
Microbial adhesion to dental composite discs in monoculture vs. co-culture (CFU/Disc) per category. MC: monoculture (dark grey). CC: co-culture (light grey).

A general comparison of adhered microorganisms to the surface of composite discs in monoculture and co-culture showed significantly lower values of *S. mutans*, *S. mitis* and *C. albicans* under the co-culture conditions (Figure 3, *p* value Mann–Whitney U < 0.001). It is relevant to notice that adhesion of *C. albicans* in co-culture to the experimental surfaces was limited by the presence of the streptococci strains, which resulted in a reduction in CFU/disc at the end of the incubation period. In addition, *S. mitis* exhibited a higher competitive ability for adhesion sites, thus reflected in a higher recovery of viable cells when compared with the other analyzed species (Figure 4).

## 4. Discussion

Based on the results, the study hypothesis was accepted, as there was a statistically significant reduction in the adhesion of the evaluated microorganisms to the modified dental composite surfaces. These findings suggest that surface characteristics play a crucial role in microbial adhesion. This supports the hypothesis that surface roughness and hydrophobicity influence microbial adherence, but more controlled topography plays a more crucial role, which is consistent with previous studies.

Biomaterials are recognized for interacting with biological systems in a compatible fashion and are employed in a diverse range of applications, including medical devices and dental products. Some surface properties, including surface chemistry and wettability, surface charge, roughness and topography, among others [22], have been credited with influencing microbial adhesion, which may be detrimental for the biomaterial and the surrounding tissues [1,2]. Recent investigations on reduction in microbial adhesion to the surface of a variety of biomaterials have made use of innovative approaches, such as surface modification, to reduce the number of microorganisms that adhere to such surfaces [23]. In the field of orthodontics, Mei et al. [24] mentioned that the interface between the orthodontic bracket, the adhesive and composite, and the enamel is a place that facilitates the adhesion of oral microorganisms that will form a biofilm. Such adhesion may be favored when an excess in the composite is found since this is a material that has exhibited a higher ability to retain oral streptococci. Bazaka et al. [16] mentioned that the adhesion of different cells to biomaterials is highly related to the surface properties of biomaterials and Cazzaniga et al. [25] described how surface roughness and chemical composition influence the microbial adhesion to materials.

The modification of the surface of different materials has demonstrated a significant reduction in the adhesion of a variety of microorganisms as controlled surfaces have demonstrated modulation of the cell response, metabolism, orientation, growth, and cell differentiation in vitro [17,26]. Surface super hydrophobicity has been considered to reduce the adhesion of bacterial species to the surface of materials [16,27]. Astasov-Frauenhoffer et al. [28] demonstrated that a surface with a low contact angle increased the formation of a biofilm. In the current investigation, the high contact angle displayed by the leaf of *C. aurea* (152.59 ± 1.96°), which classifies it as super hydrophobic [27,29], could not be observed in the experimental surfaces and showed a significant reduction in hydrophobicity. This result may be explained by the presence of protective wax coatings and hierarchical structures in the natural surface [30] that cannot be transferred to the dental composite by means of soft lithography. Furthermore, the reduction in hydrophobicity was more pronounced when the modified composite discs were coated with salivary proteins versus modified uncoated discs. Previous investigations considered that hydrophobic surface suppressed the adsorption of proteins. It has been generally considered that proteins tend to adsorb more favorably when the contact angle ranges from 60 to 90° [27,29]. In the current study, the lowest contact angle was found in the polished, saliva-coated discs, followed by the modified, saliva-coated discs. These results confirmed the results by other authors [1] and the highest adhesion of all microorganisms in monoculture occurred to the saliva-coated surfaces, being the highest to the polished, saliva-coated surfaces. According to Su et al. [22], the correlation between surface hydrophobicity and microbial adhesion is complex as many factors, including the microbial species, surface chemistry, or protein adsorption, might play influencing roles.

In the oral cavity, during the initial stage of the acquired pellicle formation on the dental surfaces, precursor salivary proteins, such as proline-rich proteins (PCP), statherins, and histatins, participate by adsorbing onto the hydroapatite crystals via calcium-binding domains. In the development stage, other salivary proteins are aggregated in globular form, thus establishing protein–protein interactions [31,32]. A study by Hannig et al. [33] showed AFM images displaying globular structures on glass slabs after 10 min of intraoral exposure. The AFM images obtained in the current work exhibited a very similar globular pattern. High molecular weight mucins are gradually incorporated increasing thickness and structural complexity until a mature stage is achieved. In this mature stage, the salivary pellicle provides specific receptors for adhesion of primary colonizers [31,32]. Proteomic studies that compare and determine the composition and function of salivary proteins from the acquired pellicle on restorative materials have shown specific patterns of protein adsorption. Reise et al. characterized the proteomic profile in situ of salivary pellicles formed on different dental composites and bovine enamel after intraoral exposure. Using mass spectrophotometry (SELDI-TOF-MS), these authors identified up to 102 different proteins in non-stimulated saliva and 46 proteins adsorbed to the acquired pellicle on dental composites and bovine enamel. Only 14 proteins showed statistically significant differences according to material type and exposure time, being α-amylase, carbonic anhydrase VI, cystatins, histatins, lysozyme, statherin, and PRP the most relevant [34]. Similarly, Hu et al. characterized the proteomic profile of the acquired pellicle on bovine enamel and different restorative materials and demonstrated that, even though dental restorative materials adsorb a fewer number of proteins compared to the bovine enamel, the protein composition is comparable. This finding suggests that biomaterials tend to be covered by a protein matrix similar to that of enamel, which might attenuate the differences in surface properties (roughness, hydrophobicity, or free surface energy) and modulate the interaction with the oral microbiota. Among the proteins that showed higher adsorption, those that exhibit higher affinity to calcium and cell–cell adhesion mediating activity and thus are involved in the stabilization of the acquired pellicle and early colonization, were more relevant [35].

*S. mitis* is a pioneer species within the oral cavity with ability to adhere rapidly and irreversibly to oral surfaces, playing a key role in the early stages of biofilm formation. Its establishment facilitates interspecific coaggregation processes with secondary colonizers, thus favoring the conformation of complex microbial communities [36]. In the current work, under monoculture conditions, *S. mitis* exhibited a higher adhesion capacity to salivary proteins-coated dental composites, followed by *S. mutans*. However, on the modified surfaces coated with salivary proteins, a significant reduction in the average number of CFUs was evident. The differences observed in the adhesion affinity on saliva-coated composite surfaces are in agreement with the results of continuous flow models that show the adhesion and proteolitic activity of *S. mitis* and *S. mutans* on surfaces treated with different protein fractions from salivary origin. *S. mitis* exhibited a strong adhesion to surfaces coated with the MUC5B mucin-enriched fraction and an aggregate of low-density proteins containing MUC7 mucin, amylase, cystatin, gp340, immunoglobulin A, lactoferrin, lysozyme, and statherin. On the other hand, *S. mutans* exhibited poor adhesion to the same proteins. Nonetheless, both species exhibited high proteolytic activity in the presence of MUC5B or low-density proteins, which suggests that the degradation of salivary components might constitute a common metabolic strategy for their survival and establishment within the oral environment. These differences in the adhesion affinity to salivary proteins associated with the acquired pellicle might be related to the colonization sequence observed in vivo, where *S. mitis* acts as primary colonizer while *S. mutans* adhere at later stages [37].

*C. albicans* is a pleomorphic yeast that exhibits the ability to transition between cellular yeast and filamentous forms. This is essential for virulence as these filamentous forms play a significant role in *C. albicans* pathogenesis [38]. As such, when *C. albicans* contacts a suitable surface, cells germinate to form hyphae and pseudohyphae. The adhesion of these morphological forms is important during biofilm development. Adhesins coordinate the adherence of pseudohyphae and hyphae, which contributes to biofilm formation and maintenance and are associated with hyphal adhesion during biofilm formation [39]. Souza et al. [40] reported that *C. albicans* promoted the formation of strong biofilms with the mitis streptococci species, including *S. mitis*, on titanium surfaces. However, the results of the current work showed a reduction in the adhesion of *C. albicans* to the modified discs in co-culture, thereby demonstrating that such modifications had a reducing effect on adhesion.

The ability of *S. mitis* to efficiently adhere to surfaces provides it with an ecological advantage by allowing competition with other microbial species for binding sites and nutrients [41]. In the current study, *S. mitis* exhibited higher competitive ability in co-culture conditions, which was evidenced by a higher number of adhered cells to experimental surfaces compared with other assessed strains. These results are in agreement with the findings of Bedoya-Correa et al. [42], who demonstrated, in vitro, that *S. mitis* and *S. mutans* exhibit better mechanisms that modulate competitive adhesion when the ability of *Streptococcus dentisani*, *S. mitis*, *S. mutans*, and *C. albicans* to form biofilms in co-culture by was assessed using the competition test.

*C. albicans* increased the adhesion to surfaces coated with saliva, which are more hydrophilic [43]. These findings are in agreement with the results reported by Thanh Nguyen et al. [44], who used hydroxyapatite microspheres and found that 93.8% of *C. albicans* cells adhered to saliva-coated microspheres as opposed to 17% to non-coated ones. Such results are evidence that the presence of saliva increases about 5.4 times the adhesive ability of *C. albicans*. *C. albicans* shows high affinity to the acquired pellicle and adhesion is produced by a strong interaction between cell-wall adhesins and the salivary pellicle [45], especially to statherin and proline-rich proteins [46,47]. The results from the current investigation showed that *C. albicans* adhere efficiently to saliva-coated smooth and modified surfaces in monoculture conditions probably due to interactions with salivary proteins adsorbed onto the surface of the dental composite. However, in co-culture conditions with *S. mitis* and *S. mutans*, a significant reduction in the adhesion of *C. albicans* to these surfaces was observed. This finding suggests a possible negative modulation in the adhesion of *C. albicans* by these streptococci strains. Consistently, a previous investigation using complex biofilms on titanium discs has reported that *C. albicans* biomass is significantly reduced when cultured with *S. mitis*, *S. gordoni*, and *S. sanguinis* when compared with monoculture conditions, where fungi adhesion is considerably higher [40]. In addition, it is important to highlight that the experimental surfaces subjected to topographic modification significantly reduced the adhesion of *C. albicans* in co-culture conditions. These results demonstrate the role of surface modification in the adhesion of *C. albicans* in mixed cultures.

A reduction in microbial adhesion to materials has been demonstrated using topographic surface modification [19,20]. The results from this investigation showed the lowest adhesion of the streptococci and the oral yeast to modified discs without salivary proteins, which agrees with the results of other studies that evaluated the reduction in adhesion to topographically modified surfaces using other microorganisms [19,20]. The presence of salivary proteins onto the modified discs, as expected, increased the microbial adhesion, but even after such increase, the adhesion to modified discs was lower than the adhesion to polished discs, with and without the presence of salivary proteins, which confirms that the changes in topography played a significant role in reducing the number of microorganisms from bacterial and mycotic origin.

*S. mutans* and *S. mitis* have been described as hydrophilic species and *S. mitis* tends to adhere preferably to hydrophilic surfaces [48], while *S. mutans* is slightly more hydrophobic and shows a tendency to adhere to surfaces that are not coated with saliva [49], though microbial adhesion to the surface of materials is a complex process that is dependent on many factors, both from the species and from the materials, and must be analyzed considering these many factors [48]. However, the results of this study showed more adhesion of *S. mutans* to more hydrophilic surfaces. According to the results of the current investigation, the evaluated microorganisms exhibited a preference to adhere to hydrophilic surfaces, with or without surface modification, which may be explained by the protein composition of the saliva. Therefore, the results from the current work highlight the function of salivary proteins and their influence in the regulation of microbial growth on dental composites modified following a biomimetic approach. Specific interactions between protein molecules, the topographic modification, and the oral microorganisms play a definitive role in the reduction in microbial adhesion and, consequently, on biofilm formation. Understanding these interactions is essential to design biomimetic strategies of surface modification oriented to the microbiological control in dental biomaterials used in daily practice.

As for surface roughness, the polished discs without saliva coating demonstrated to be the smoothest surface, while the modified discs without saliva coating exhibited the highest roughness surface. This increase in roughness from polished to modified surfaces confirms the results reported in another study, even though a different material was used [50]. Coating the polished discs with saliva increased their surface roughness, which agrees with results by different authors [51,52,53]. It has been suggested that the presence of saliva may cause an erosion of the composite surface derived from the debonding of the filler from the matrix or by fragmentation of inorganic particles [52,54]. On the other hand, coating the modified discs with saliva reduced its surface roughness, which may be explained by the creation of a lubrication layer that might fill irregularities on the surface, thus making it smoother [55]. Even though the uncoated modified discs showed the highest roughness, microbial adhesion exhibited the lowest numbers, which confirms that topographic modification might play a more important role than roughness when analyzing microbial adhesion. This reduction in microbial adhesion to topographically modified surfaces, which is the opposite of what it is observed on rough surfaces, may be explained by the fact that even though surface roughness increases the surface area and creates retention sites where microorganisms may endure and thrive, topographical patterns may affect the contact available area, thus limiting access to flat regions for microorganisms to adhere, impose physical stress on their cell membranes [22,56] or act as obstacles or barriers for microorganisms to organize in engineered topographies [57].

Even though some study limitations were present, including the number of microbial species, materials, and topographic features on evaluated surfaces, these results encourage further investigation into the behavior of different microorganisms when in the presence of topographical features on the surface of biomaterials and open new possibilities to continue understanding how to reduce the adhesion of bacteria, and other microorganisms, to the surface of biomedical materials. Future works should increase the sample size and number of repetitions, analyze more complex microbial communities and the role of each salivary protein regarding microbial adhesion in a more detailed fashion. In addition, surfaces with higher contact angles (>90°) should also be considered to analyze the interaction of these microbials with such surfaces.

## 5. Conclusions

Considering the limitations of the current investigation, the topographic modification of the surface of a dental biomaterial reduced the adhesion of different microorganisms, namely bacteria and yeasts. Topographic surface patterning played a more relevant role at reducing the adhesion of the assessed microorganisms than the evaluated surface characteristics (roughness and hydrophobicity). In addition, the adhesion of *S. mutans*, *S. mitis*, and *C. albicans* in monoculture and co-culture on saliva-coated surfaces was higher than on topographically modified surfaces. The promising results obtained in this work encourage further investigation to advance the knowledge on the behavior of microorganisms when in contact with topographic patterns and elucidate the mechanisms by which topographic surface modification of biomaterials reduce the adhesion of microorganisms with the objective of finding alternative, synergistic, non-chemical methods to reduce microbial adhesion and biomaterials failure.

## Figures and Tables

**Table 1:** Microbial adhesion to the composite discs in monoculture (CFU/Disc).

Strains	Categories	CFU/Disc Mean ± SD	Minimum–Maximum	*p* Value
*S. mutans*	L-SSal ^c^	2.08 × 10^6^ ± 1.25 × 10^6^	6.00 × 10^5^–4.50 × 10^6^	<0.001
	L-Sal ^b^	6.25 × 10^6^ ± 3.44 × 10^6^	1.00 × 10^6^–1.10 × 10^7^	
	P-SSal ^a^	1.01 × 10^6^ ± 8.40 × 10^5^	7.00 × 10^4^–2.10 × 10^6^	
	P-Sal ^a^	6.87 × 10^5^ ± 6.28 × 10^5^	5.00 × 10^4^–1.60 × 10^6^	
*S. mitis*	L-SSal ^a^	6.20 × 10^6^ ± 5.51 × 10^6^	1.60 × 10^6^–1.80 × 10^7^	<0.001
	L-Sal ^b^	2.17 × 10^7^ ± 1.22 × 10^7^	3.00 × 10^6^–4.10 × 10^7^	
	P-SSal ^c^	5.53 × 10^6^ ± 4.96 × 10^6^	9.00 × 10^5^–1.30 × 10^7^	
	P-Sal ^a^	6.30 × 10^6^ ± 5.58 × 10^6^	1.00 × 10^6^–1.80 × 10^7^	
*C. albicans*	L-SSal	8.50 × 10^5^ ± 3.02 × 10^5^	4.00 × 10^5^–1.20 × 10^6^	0.100
	L-Sal	1.10 × 10^6^ ± 7.70 × 10^5^	3.00 × 10^5^–2.60 × 10^6^	
	P-SSal	4.37 × 10^5^ ± 2.32 × 10^5^	2.00× 10^5^–9.00 × 10^5^	
	P-Sal	8.12 × 10^5^ ± 5.51 × 10^5^	1.00 × 10^5^–1.90 × 10^6^	

Kruskal–Wallis H test, *p* < 0.05. CFU/Disc: Colony Forming Units per disc; SD: standard deviation; L-SSal: polished, uncoated; L-Sal: polished, coated with salivary proteins; P-SSal: modified, uncoated; P-Sal: modified, coated with salivary proteins. a, b, c: experimental groups with different keys show significant differences (Games-Howell *p* < 0.05). A colony-forming unit (CFU) is a viable cell or group of cells forming a visible colony on a growth medium. CFUs are used as a measure of viable microbial numbers that can be counted and express a quantitative measure of microbial load in a sample [21].

**Table 2:** Microbial adhesion to dental composite discs in co-culture (CFU/disc).

Strains	Categories	CFU/Disc Median (IQR)	Minimum–Maximum	*p* Value (Kruskal–Wallis H)
*S. mutans*	L-SSal ^a^	1.0 × 10^2^ (1.0 × 10^2^–4.00 × 10^2^)	2.00 × 10^1^–1.00 × 10^3^	<0.001
	L-Sal ^b^	1.15 × 10^3^ (1.00 × 10^3^–3.50 × 10^3^)	8.00 × 10^2^–7.00 × 10^3^	
	P-SSal ^a^	1.00 × 10^2^ (3.50 × 10^1^–2.00 × 10^2^)	1.00 × 10^1^–6.00 × 10^2^	
	P-Sal ^a^	1.25 × 10^2^ (3.50 × 10^1^–6.50 × 10^2^)	2.00 × 10^1^–1.20 × 10^3^	
*S. mitis*	L-SSal	1.24 × 10^5^ (1.70 × 10^4^–2.45 × 10^5^)	3.00 × 10^3^–3.00 × 10^5^	0.661
	L-Sal	8.00 × 10^4^ (1.50 × 10^3^–5.80 × 10^5^)	1.20 × 10^3^–7.00 × 10^5^	
	P-SSal	2.80 × 10^4^ (1.35 × 10^4^–4.85 × 10^4^)	9.00 × 10^3^–1.50 × 10^5^	
	P-Sal	9.50 × 10^4^ (1.15 × 10^4^–1.65 × 10^5^)	1.10 × 10^3^–3.40 × 10^5^	
*C. albicans*	L-SSal ^a^	1.00 × 10^2^ (4.5 × 10^1^–2.00 × 10^2^)	3.00 × 10^1^–2.00 × 10^2^	<0.001
	L-Sal ^a^	2.00 × 10^2^ (1.00 × 10^2^–1.0 × 10^3^)	1.00 × 10^2^–2.00 × 10^3^	
	P-SSal ^b^	1.50 × 10^1^ (1.00 × 10^1^–3.50 × 10^1^)	1.00 × 10^1^–2.00 × 10^2^	
	P-Sal ^b^	1.00 × 10^1^ (1.00 × 10^1^–2.50 × 10^1^)	1.00 × 10^1^–4.00 × 10^2^	

Kruskal–Wallis H test, *p* < 0.05. CFU/Disc: Colony Forming Units per disc; IQR: interquartile range; L-SSal: polished, uncoated; L-Sal: polished, coated with salivary proteins; P-SSal: modified, uncoated; P-Sal: modified, coated with salivary proteins. a, b: experimental groups with different keys show significant differences (Games-Howell *p* < 0.05).

## Data Availability

The data presented in this study are available from the corresponding author upon request.

## Abbreviations

The following abbreviations are used in this manuscript:

AP	Acquired Pellicle
PDMS	Poly (Dimethyl Siloxane)
CFU	Colony Forming Units
